# P-547. Single-Cell Transcriptomic Analysis of Severe Acute Encephalopathy/Encephalitis Associated with SARS-CoV-2 Reveals HSPA1A and HSPB1 as Potential Markers

**DOI:** 10.1093/ofid/ofaf695.762

**Published:** 2026-01-11

**Authors:** Takako Suzuki, Yoshitaka Sato, Motomasa Suzuki, Yuto Fukuda, Ken-ichi Iwata, Makoto Yamaguchi, Yoshiki Kawamura, Tetsushi Yoshikawa, Yoshiyuki Takahashi, Hiroshi Kimura, Jun-ichi Kawada, Yuka Torii

**Affiliations:** Nagoya University Graduate School of Medicine, Nagoya, Aichi, Japan; Department of Virology, Nagoya University Graduate School of Medicine, Nagoya, Aichi, Japan; Department of Pediatric Neurology, Aichi Children's Health and Medical Center, Obu, Aichi, Japan; Nagoya University Graduate School of Medicine, Nagoya, Aichi, Japan; Department of Pediatrics, Nagoya University Graduate School of Medicine, Nagoya, Aichi, Japan; Department of Pediatrics, Nagoya University Graduate School of Medicine, Nagoya, Aichi, Japan; Fujita Health University Okazaki Medical Center, Okazaki, Aichi, Japan; Fujita Health University School of Medicine, Toyoake, Aichi, Japan; Department of Pediatrics, Nagoya University Graduate School of Medicine, Nagoya, Aichi, Japan; Department of Virology, Nagoya University Graduate School of Medicine, Nagoya, Aichi, Japan; Fujita Health University School of Medicine, Toyoake, Aichi, Japan; Nagoya University Graduate School of Medicine, Nagoya, Aichi, Japan

## Abstract

**Background:**

Acute encephalopathy/encephalitis (AE) associated with SARS-CoV-2 has been increasingly reported since the emergence of the Omicron variant. Some patients developed acute fulminant cerebral edema (AFCE) or hemorrhagic shock encephalopathy syndrome (HSES) and had poor prognoses; however, the underlying pathogenesis of these conditions remains unclear. In this study, we performed single-cell RNA sequencing (scRNA-seq) on a patient with SARS-CoV-2-associated AE diagnosed with AFCE/HSES and compared the findings with those from patients with mild AE and febrile seizures caused by other pathogens. We also compared these cases with pediatric patients with COVID-19 without neurological complications.

**Methods:**

Four pediatric patients were enrolled: one with SARS-CoV-2-associated AFCE/HSES, two with mild AE of different etiologies (influenza A and Epstein–Barr virus), and one with febrile seizures associated with HHV-6. Peripheral blood mononuclear cell (PBMC) samples were collected from each patient during the acute and convalescent phases. Single-cell RNA libraries were prepared using the 10x Chromium platform. The sequences were analyzed using Cell Ranger and Seurat. Publicly available scRNA-seq PBMC data for pediatric patients with COVID-19 were used for comparison.

**Results:**

A total of 109,940 cells were analyzed in this study. In the acute phase of SARS-CoV-2-associated AFCE/HSES, the B-cell fraction was markedly increased. Enrichment analysis of upregulated genes in B cells showed activation of pathways associated with protein stabilization (GO:0050821), with particularly significant upregulation of HSPA1A and HSPB1. The gene expression levels of these heat shock proteins were low during the convalescent phases of SARS-CoV-2-associated AFCE/HSES, mild AE, and COVID-19. ELISA measurements of HSPA1A and HSPB1 in plasma and serum of the three AE cases revealed a marked increase during the SARS-CoV-2-associated AFCE/HSES acute phase.

**Conclusion:**

Our findings suggest that HSPA1A and HSPB1 are potential markers of SARS-CoV-2-associated severe AE, although further studies are needed to validate their clinical utility.

**Disclosures:**

All Authors: No reported disclosures

